# Combined Application of Biofertilizers and Inorganic Nutrients Improves Sweet Potato Yields

**DOI:** 10.3389/fpls.2017.00219

**Published:** 2017-03-13

**Authors:** Ruth W. Mukhongo, John B. Tumuhairwe, Peter Ebanyat, AbdelAziz H. AbdelGadir, Moses Thuita, Cargele Masso

**Affiliations:** ^1^Department of Agricultural Production, School of Agricultural Sciences, Makerere UniversityKampala, Uganda; ^2^International Institute of Tropical AgricultureKampala, Uganda; ^3^Soil Microbiology Laboratory, International Institute of Tropical AgricultureIbadan, Nigeria; ^4^International Institute of Tropical AgricultureNairobi, Kenya

**Keywords:** arbuscular mycorrhizal fungi, drought stress, root colonization, nutrient concentration, sweet potato, yield gap

## Abstract

Sweet potato [*Ipomoea batatas* (L) *Lam*] yields currently stand at 4.5 t ha^−1^ on smallholder farms in Uganda, despite the attainable yield (45–48 t ha^−1^) of NASPOT 11 cultivar comparable to the potential yield (45 t ha^−1^) in sub-Saharan Africa (SSA). On-farm field experiments were conducted for two seasons in the Mt Elgon High Farmlands and Lake Victoria Crescent agro-ecological zones in Uganda to determine the potential of biofertilizers, specifically arbuscular mycorrhizal fungi (AMF), to increase sweet potato yields (NASPOT 11 cultivar). Two kinds of biofertilizers were compared to different rates of phosphorus (P) fertilizer when applied with or without nitrogen (N) and potassium (K). The sweet potato response to treatments was variable across sites (soil types) and seasons, and significant tuber yield increase (*p* < 0.05) was promoted by biofertilizer and NPK treatments during the short-rain season in the Ferralsol. Tuber yields ranged from 12.8 to 20.1 t ha^−1^ in the Rhodic Nitisol (sandy-clay) compared to 7.6 to 14.9 t ha^−1^ in the Ferralsol (sandy-loam) during the same season. Root colonization was greater in the short-rain season compared to the long-rain season. Biofertilizers combined with N and K realized higher biomass and tuber yield than biofertilizers alone during the short-rain season indicating the need for starter nutrients for hyphal growth and root colonization of AMF. In this study, N0.25PK (34.6 t ha^−1^) and N0.5PK (32.9 t ha^−1^) resulted in the highest yield during the long and the short-rain season, respectively, but there was still a yield gap of 11.9 and 13.6 t ha^−1^ for the cultivar. Therefore, a combination of 90 kg N ha^−1^ and 100 kg K ha^−1^ with either 15 or 30 kg P ha^−1^ can increase sweet potato yield from 4.5 to >30 t ha^−1^. The results also show that to realize significance of AMF in nutrient depleted soils, starter nutrients should be included.

## Introduction

Sweet potato [*Ipomoea batatas* (L) Lam] is an important staple food crop in many parts of the tropics. Globally, it is ranked the seventh most important food crop after wheat, rice, maize, Irish potato, barley, and cassava (Mwanga et al., 2001). It is a substantial source of carbohydrate and beta-carotene (FAO, 2002). In Africa, sweet potato is the second most important root crop after Irish potato and it is produced mainly in East African countries around Lake Victoria. It is a staple food crop in Uganda, where it is among the priority food security crops (Aritua and Gibson, 2002; Kapinga et al., 2007). Productivity of sweet potato is constrained by poor fertility especially low potassium (K), phosphorus (P), nitrogen (N), sulfur (S), and micronutrients (copper, zinc, iron, manganese, molybdenum, boron, chlorine, and nickel) (Bourke, 2005, 2009; Bailey et al., 2008; Kirchlof, 2009; Taraken et al., 2010; Uwah et al., 2013). The estimated nutrient removal by sweet potato from soil is 100, 90, and 200 kg ha^−1^ of N, P, and K, respectively, which may result to 20–40 t ha^−1^ of marketable roots depending on cultivar and management (Traynor, 2005). In highly nutrient depleted soils of sub-Saharan Africa (SSA), balanced nutrition of N, P, and K is required to enhance crop yield (O'Sullivan et al., 1997).

In Uganda, sweet potato tuber yields on smallholder farms stand at an average of 4.5 t ha^−1^ (CIP, 2006), which is 10% of the attainable yield (45–48 t ha^−1^) of the cultivar Namulonge Sweet Potato 11 (NASPOT 11; Mwanga et al., 2011) and the potential yield (45 t ha^−1^) in SSA (CIP, 2006). NASPOT 11 has long-elliptic storage root shape when grown in light soils, has high dry matter content (~34%) which translates into increased yield and good to excellent consumer acceptance, depending on growth conditions. In terms of resistance to diseases such as sweet potato virus disease (SPVD) and *Alternaria bataticola* blight, the cultivar is superior to previously released cultivars (Mwanga et al., 2011). The low on-farm sweet potato yields are recorded in other SSA countries like Kenya and Ethiopia with 9.5 and 7.7 t ha^−1^, respectively, among others (PRAPACE, 2003). Although, yields are substantially below their potential, experimental yields of more than 25 t ha^−1^ have been obtained with the use of fertilizers (MAAIF, 1992). Osiru et al. (2009) showed significant variation (*p* < 0.05) at Kachwekano Agricultural Research and Development Centre in south-western Uganda (41.10 tons per hectare) than at Namulonge Agricultural Research Station in central Uganda (16.08 tons per hectare) and at Serere Animal Agricultural Research Station in eastern Uganda (14.75 tons per hectare) across genotypes and seasons. While the germplasm constraint has been fairly addressed through breeding, the abiotic and edaphic constraints, especially soil fertility, still limits potential yield of sweet potato (Pender et al., 2004). Possible interventions to alleviate soil fertility limitation include application of inorganic fertilizers, organic fertilizers and biofertilizers. Biofertilizers are increasingly being included in integrated soil fertility management (ISFM) programs in Asia and Africa (Vanlauwe et al., 2010). Intensified use of biofertilizers such as arbuscular mycorrhizal fungi (AMF) is emerging as an environmentally-friendly alternative soil fertility management practice with potential to increase and cheaply sustain crop yields compared with continuous application of inorganic fertilizers alone (Sharma et al., 2013). Introducing AMF to soils which already contain AMF could be beneficial since the carrying capacity of some agricultural soils may be low yet high spore densities are required to increase the volume of hyphae in the soil and the percentage of roots colonized (Rosendahl, 2008). Several benefits of AMF have been reported including improved plant survival and acclimatization, increased growth and nutrient uptake (especially P, Zn, Mn, Mg, Cu, K, and N), increased crop yields (Estaun et al., 2002; Wang et al., 2008; Kapulnik et al., 2010; Ortas, 2010; Kavoo-Mwangi et al., 2013) and improved water use efficiency (WUE; Auge, 2001). These have been reported for plants and crops like olive (Porras-Soriano et al., 2010), banana (Kavoo-Mwangi et al., 2013, 2014), turmeric (Radhika and Rodrigues, 2010) and cassava (Ceballos et al., 2013), and sweet potato (Abdel-Razzak et al., 2013).

Abdel-Razzak et al. (2013) reported that integrating AMF inoculum with superphosphate fertilizer under the recommended P level (100% P_2_O_5_) enhanced root productivity and quality than when the treatments were applied singly. However, despite the Abuja declaration of 2006 to increase inorganic fertilizer use to at least 50 kg nutrient per hectare, little impact on this has been realized in most parts of SSA due to financial constraints experienced by smallholder farmers. Moreover, a great proportion of applied P fertilizer is not available to plants due to strong fixation of P on iron (Fe), manganese (Mn), and aluminum (Al) oxides (Bünemann et al., 2004; Cardoso and Kuyper, 2006) in most tropical soils. Therefore, we hypothesized that the introduced AMF will improve P availability through solubilization of the P present in the soils, and moisture mobilization leading to increased growth and yield of sweet potato. This prompted the investigation on how to reduce inorganic P fertilizer use by integrating N and K with biofertilizer in increasing sweet potato yields. This was evaluated by comparing AMF to varying P rates in a Ferralsol and Rhodic Nitisol soil in Uganda, and it was predicted that there will be slight differences in the performance of the treatments in the two soils due to their almost identical low to medium fertility levels.

## Materials and methods

### Description of experimental sites

**Figure 1:**
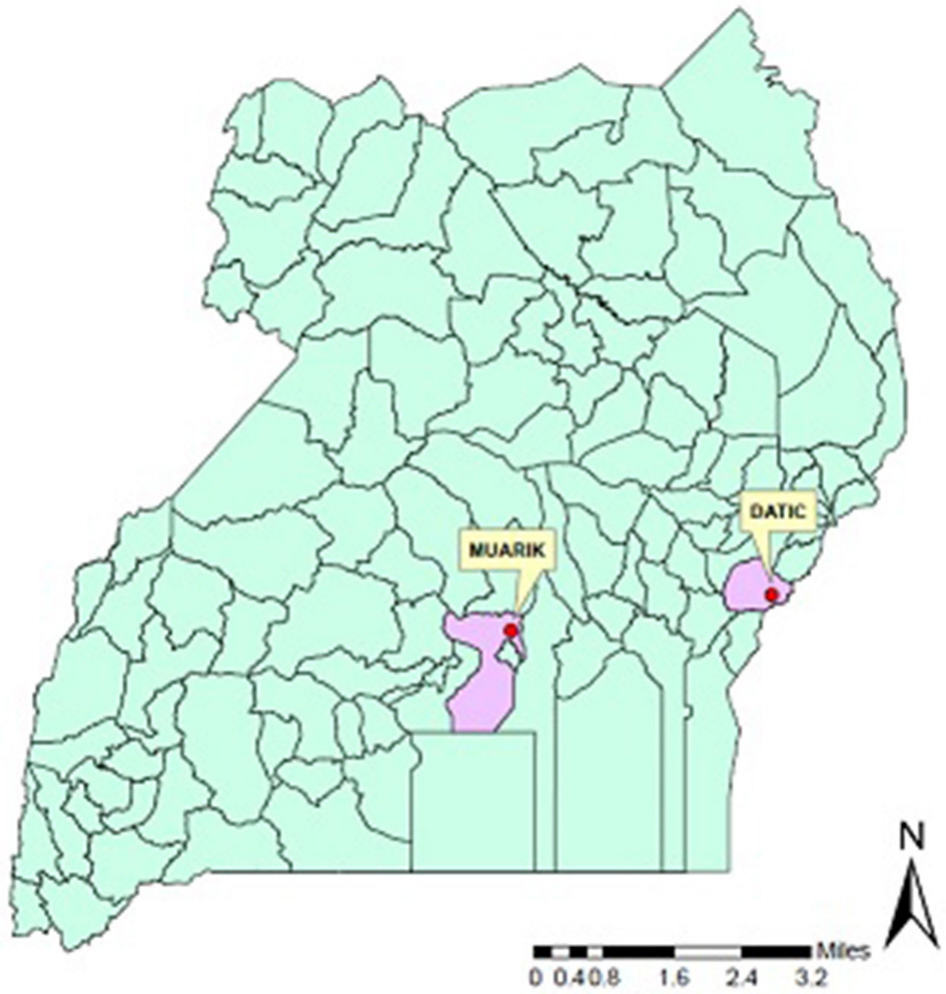
**A map of Uganda showing the location of DATIC and MUARIK experimental sites**.

On-station experiments were conducted in the Mt Elgon High Farmlands and Lake Victoria Crescent located in eastern and central Uganda, respectively for two seasons (Figure 1). In eastern Uganda, the trial was conducted at the District Agricultural Training Centre (DATIC) in Tororo located at 0° 40′ 57″ N and 34° 10′ 45″ E. The annual mean rainfall is 1100 mm distributed bimodally (March to June and September to November) and the monthly mean temperature is 25°C. The dry season is mostly pronounced during December to February. The soil at this site is mainly sandy loam classified as Ferralsol. In central Uganda, the experiment was done at the Makerere University Agricultural Research Institute, Kabanyolo (MUARIK) in Wakiso District, located at 0^0^ 28′N and 32° 37′E. The soil at this site is of sandy clay texture and classified as Rhodic Nitisol. The area also receives bimodal rainfall during the months of February to May and August to December with an annual mean of 1160 mm. The station's monthly mean temperature is 24.5°C (Yost and Eswaran, 1990).

### Soil sampling and analysis

Ten sub-samples were collected in March and September, 2014 using an auger at a depth of 0–20 cm in each site following a zigzag pattern, and soils were homogeneously mixed and 1 kg composite sample obtained through quartering. Each soil sample was air-dried, sieved through 2 mm sieve, homogenized and analyzed for pH (in 1:2.5 H_2_O), total carbon, total and available P, exchangeable cations (Ca^2+^, Mg^2+^, and K^+^) and texture in Makerere University Soil and Plant Analytical Laboratory. All analyses were performed following routine procedures. Soil pH was measured in a soil-water solution at a ratio of 1:2.5 (w/v) using a pH meter (Mettler-Toledo, AG 8603; Rhoades, 1982). Total N was determined using Kjeldahl digestion method (Anderson and Ingram, 1989a). Available P was extracted using Bray 1 method (Anderson and Ingram, 1989b) and read using a spectrophotometer (Jenway, 6405 UV/Vis). Exchangeable bases were extracted in ammonium acetate and measured using a flame-photometer (K^+^) (Jenway, Essex CM6 3LB) and atomic absorption spectrophotometer (Ca^2+^, Mg^2+^) (Anderson and Ingram, 1993b; Jenway, 6405 UV/Vis). Total carbon was determined by wet oxidation and titration (Anderson and Ingram, 1993a). Soil texture was determined using a Bouyoucos (Gallenkamp Bouyoucos) method (Bouyoucos, 1962). The soil water holding capacity was determined using the soil texture hydraulic properties calculator. AMF spores were extracted from the test soils and AMF based biofertilizers using Jenkins (1964) procedure as modified by Ingleby (2007) and identified according to their morphotypes on the basis of spore morphology and subcellular characters with reference to the original species descriptions in INVAM. (2017) and Schenck and Perez (1990).

### Land preparation and experimental establishment

Land selected at MUARIK had been used as a pasture paddock for almost 10 years and later converted to crop field, mainly maize for 1 year. At DATIC, the field had been under maize and cassava production for 2 years. All the experimental sites had not been inoculated with AMF previously. Land was ploghed using a tractor and animal-drawn plogh at MUARIK and DATIC, respectively, but mounds of about 0.5 m height and 1 m length were manually heaped. A common practice of an inter-row spacing of 1 m and intra-row spacing of 1 m was used giving a total of 30 mounds within a plot size of 5 m by 6 m and planted with NASPOT 11 sweet potato cultivar. This is one of the recently released high yielding varieties and resistant to pests and diseases. It also produces high biomass which can be fed to livestock (Mwanga et al., 2011). Sweet potato tip cuttings about 25 cm were planted at an angle of 45° with two thirds of the vine under the soil for proper establishment. The first and the second season experiments were established in April 2014 and October 2014, respectively. The experiment consisted of 10 treatments (Table 1) arranged in a randomized complete block design (RCBD) with four replicates. The study sites were blocked across gentle slopes to obtain homogenous plots in the blocks. Biofertilizers were applied per mound (1 m^2^) at a rate of 50 g Rhizatech and 1.3 g Symbion vam plus. Biofertilizers were applied to soil based on the manufacturers' recommendations. Rhizatech is produced by Dudutech Kenya Ltd while Symbion vam plus is produced by T. Stanes & Company Ltd and marketed by Osho Chemical Industries Ltd. The biofertilizers were tested since they were the only AMF products available in the nearby market and they are recommended for all AMF host crops. Inorganic P fertilizer (Triple Super Phosphate) was applied at five rates of 0, 15, 30, 45, and 60 kg ha^−1^ representing 0, 25, 50, 75, and 100% of the full rate, respectively. Nitrogen and K were applied at blanket rates of 90 and 100 kg ha^−1^, respectively. All the P fertilizer and a third of the N (Urea) and K (Muriate of Potash) fertilizers were applied at planting and top-dressed with the two-thirds of the N and K fertilizers at 2 months after planting (2 MaP). The plots were kept free of weeds by manual weeding. The crop was managed for 4 months before harvesting.

**Table 1:** **Description of treatments applied in the experiment**.

**Treatment**	**Composition**	**Rate**
1. Control	None	None
2. N0PK	Nitrogen and Potassium	90 kg N and 100 kg K ha^−1^
3. N0.25PK	Nitrogen, Phosphorus, and Potassium	90 kg N, 15 kg P and 100 kg K ha^−1^
4. N0.5PK	Nitrogen, Phosphorus, and Potassium	90 kg N, 30 kg P and 100 kg K ha^−1^
5. N0.75PK	Nitrogen, Phosphorus, and Potassium	90 kg N, 45 kg P and 100 kg K ha^−1^
6. NPK	Nitrogen, Phosphorus, and Potassium	90 kg N, 60 kg P and 100 kg K ha^−1^
7. Rhizatech	(*Glomus mosseae, G. intraradices, G. etunicatum*, and *G. claroideum*) (4 spores g^−1^)	500 kg Rhizatech ha^−1^
8. Rhizatech+NK	(*Glomus mosseae, G. intraradices, G. etunicatum*, and *G. claroideum*) (4 spores g^−1^), Nitrogen and Potassium	500 kg Rhizatech ha^−1^, 90 kg N and 100 kg K ha^−1^
9. Symbion vam plus	*Glomus* and *Gigaspora* spp. of AMF (2 spores g^−1^) and *Bacillus megaterium* var. phosphaticum (4^*^10^4^ CFUs g^−1^)	13 kg Symbion vam plus ha^−1^
10. Symbion vam plus+NK	*Glomus* and *Gigaspora* spp. of AMF (2 spores g^−1^) and *Bacillus megaterium* var. phosphaticum (4^*^10^4^ CFUs g^−1^), Nitrogen and Potassium	13 kg Symbion vam plus ha^−1^, 90 kg N and 100 kg K ha^−1^

### Data collection

Plants in each plot, excluding the border mounds, were assessed for AMF root colonization, nutrient recovery, biomass accumulation and tuber yield. Destructive sampling of nine plants from three mounds per treatment per replicate was done at two and four MaP to determine AMF root colonization, while biomass accumulation, nutrient recovery and tuber yields were determined at four MaP.

#### AMF root colonization intensity

Sub-samples of roots were processed for mycorrhiza colonization according to procedures by Koske and Gemma (1989). Into each bottle 2.5% potassium hydroxide (KOH) was added before heating in the oven at 70°C for 1 h. The KOH was poured off and the roots rinsed to remove KOH. Alkaline hydrogen peroxide (60 ml of 28–30% NH_4_OH, 90 ml of 30% H_2_O_2_, and 840 ml distilled water) was then added and roots left for 1 h to remove the phenolic substances. Alkaline hydrogen peroxide was poured off, the roots thoroughly rinsed with tap water and 1% hydrochloric acid (HCl) added and left for 1 h. After pouring off HCl 0.05% Trypan blue was added and the roots placed in the oven for 1 h. De-staining solution (500 mL glycerol, 450 mL of distilled water, and 50 mL of 1% HCl) was added. Slides were prepared with 30 pieces of roots each 1 cm long then examined under a compound microscope at magnification x40. The percentage of each piece covered by arbuscules, vesicles, hyphae and intraradical spores was assessed to determine the intensity of AM fungi colonization (Equation 1; McGonigle et al., 1990).

(1) $$\begin{array}{l} \text{Colonization intensity}\left(\%\right)=\\ 100\left(\frac{\left(\text{n}1\times 10\right)+\left(\text{n}2\times 30\right)+\left(\text{n}3\times 50\right)+\left(\text{n}4\times 70\right)+\left(\text{n}5\times 90\right)}{\text{N}}\right) \end{array}$$

A scale of 1–5 was used

Where 1 = 0–20; 2 = 21–40; 3 = 41–60; 4 = 61–80 and 5 = 81–100% root colonization.

n1, n2—n5 indicate the number of fine roots with an intensity of 1, 2, —5.

N = number of fine roots observed.

#### Nutrient recovery

The oven-dried vine samples were ground in a ball mill and analyzed for total P, K, and Zn. Emphasis was put on P and Zn nutrients because AMF has frequently been reported to increase their uptake and concentration in plant tissue, while K is required in large quantities by sweet potato. The concentration of total P was assessed after wet digestion of air-dried ground plant samples with a mixture of concentrated sulphuric acid (H_2_SO_4_) and selenium powder and salicylic acid and measured using a spectrophotometer (Jenway, 6405 UV/Vis). Potassium was assessed from the digested sample using a flame photometer (Jenway Ltd, Felsted, Dunmow, Essex CM6 3LB), while Zn was assessed using atomic absorption spectrophotometer (Savant AAA series version 3.02, GBC Scientific Equipment) following digestion (Okalebo et al., 2002).

#### Biomass accumulation and tuber yield

Soil was carefully removed from the tuber and non-tuber roots. Fresh weights of all vines and roots were weighed in the field using a balance (Terraillon, Hanson H1040 of 50 kg and accuracy ± 0.025 kg). Sub-samples of tuber, non-tuber and vines were oven dried at 60°C for 72 h and dry matter weighed with a precision balance (Phillips Harris, Shemstone England, 4.5 kg and accuracy ±0.01 g).

### Data analysis

Root colonization, nutrient concentration, biomass accumulation and yield data in individual sites were fit in a general linear model and subjected to analysis of variance using Statistical Analysis Software (SAS), version 9.4 for analysis of variance. Pre-ANOVA assessment included the test of normality of the data. Means were separated using Tukey HSD test. Treatment contrasts were used to compare differences between pairs and groups of treatments and they included: 1 vs. 2,3,4,5,6 = Control vs. NPK treatments; 1 vs. 7,8,9,10 = Control vs. Biofertilizer treatments; 2 vs. 3,4,5,6 = NK vs. NPK; 2 vs. 7,8,9,10 = NK vs. Biofertilizer treatments; 3,4,5,6 vs. 7,8 = NPK vs. Rhizatech treatments; 3,4,5,6 vs. 7,8 = NPK vs. Symbion vam plus treatments; 7,8 vs. 9,10 = Rhizatech treatments vs. Symbion vam plus treatments; 7 vs. 8 = Rhizatech vs. Rhizatech+NK and 9 vs. 10 = Symbion vam plus vs. Symbion vam plus+NK. Simple linear regression was conducted using SAS REG procedure to determine the relationship between AMF root colonization and P, K and Zn concentration, VDW, RDW, and yield in the sweet potato vines.

## Results

### Soil characterization

The total carbon content was high and low for the Rhodic Nitisol and Ferralsol soil, respectively. The total N for the Ferralsol was slightly low, while that of the Rhodic Nitisol was within the medium fertility range. Available P levels for both soils were very low considering a moderate range of 12-20 mg per kg of soil (Cook, 1988). The exchangeable bases were sufficient for crop production except for calcium (Ca) in the Ferralsol. There was a moderate population density of indigenous AMF (4–5 spores g^−1^ of soil) in both sites (Table 2) which was comparable to the spore densities in the biofertilizers (4 spores g^−1^ of product) used in this study (Table 1).

**Table 2:** **Selected physical and chemical properties and arbuscular mycorrhizal fungi characterization of experimental soils before planting**.

**Soil property**	**Units**	**Ferralsol (Mean ±*SD*)**	**Rating**	**Rhodic Nitisol (Mean ±*SD*)**	**Rating**
pH (in H_2_0)		5.82 ± 0.07	Moderate acidity^a^	5.57 ± 0.12	Moderate acidity^a^
Total carbon (T.C)	%	1.25 ± 0.08	Low^a^	3.21 ± 0.29	Medium^a^
Total N	%	0.11 ± 0.01	Low^a^	0.14 ± 0.002	Medium^a^
Total P	mg kg^−1^	775.00 ± 50		753.00 ± 2.50	
Available P	mg kg^−1^	3.76 ± 1.80	Low^b^	1.54 ± 0.02	Low^b^
Exchangeable Ca	Cmol(+) kg^−1^	2.38 ± 0.63	Low^a^	2.50 ± 0.03	Medium^a^
Exchangeable Mg	Cmol(+) kg^−1^	0.79 ± 0.21	High^a^	0.83 ± 0.09	High^a^
Exchangeable K	Cmol(+) kg^−1^	0.23 ± 0.02	Medium^a^	0.29 ± 0.004	Medium^a^
Sand	%	73 ± 0.50		51 ± 0.50	
Silt	%	16 ± 0.50		12 ± 0.50	
Clay	%	11 ± 0.50		37 ± 0.50	
Textural class		Sandy loam		Sandy clay	
Water holding capacity	cm^3^ water/cm^3^ soil	0.19 ± 0.01		0.31 ± 0.01	
AMF population (*Glomus, Gigaspora, Scutellospora*, and *Acaulospora* spp.)	spores/g soil	5 ± 0.50		4 ± 0.20	

*Analysis was done in duplicates. Rating was done according to Okalebo et al. (2002)^a^ and Cook (1988)^b^*.

### Rainfall distribution across sites and seasons

**Figure 2:**
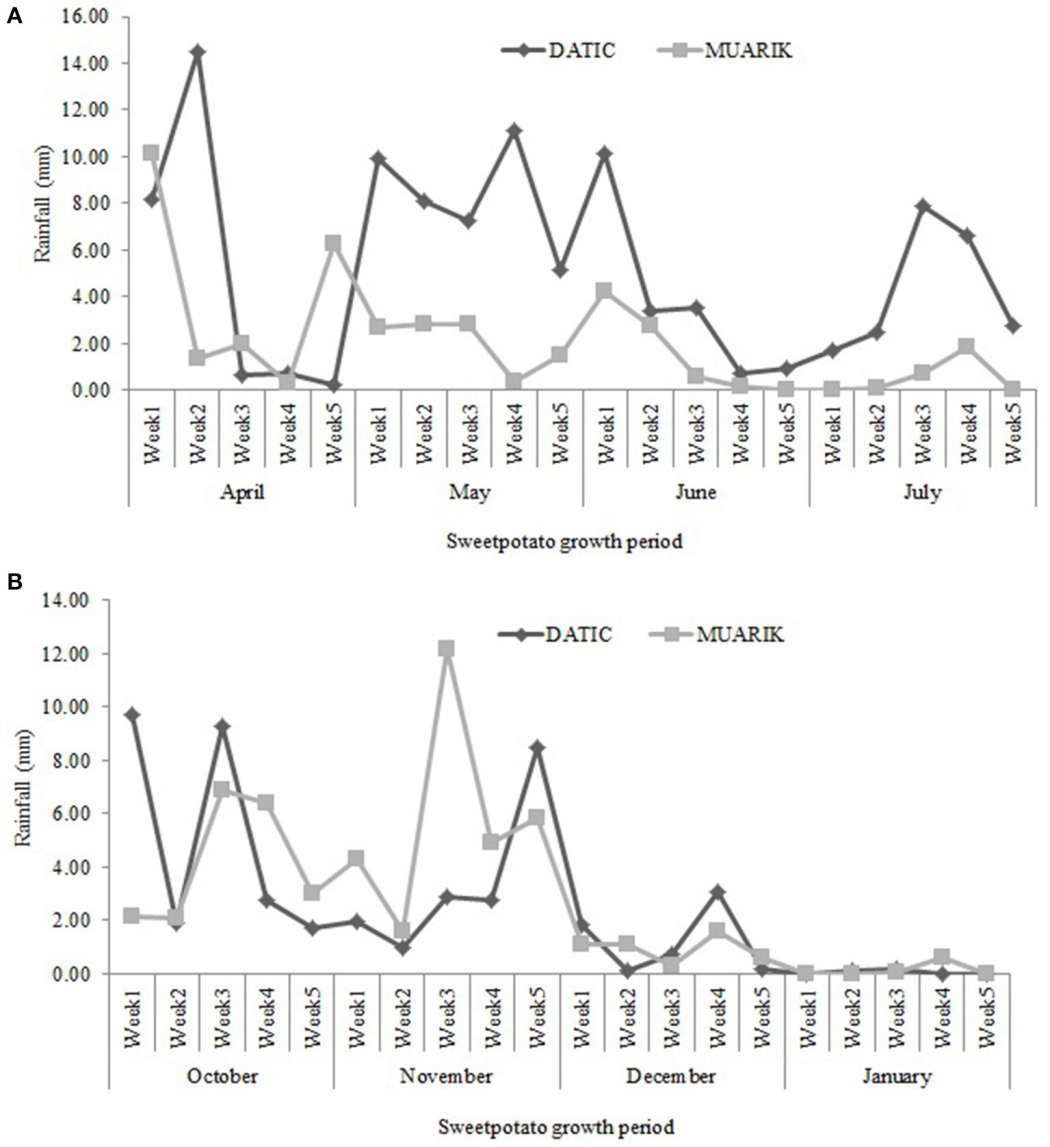
**Rainfall distribution at DATIC and MUARIK sites for the (A)** long-rain season (April to July, 2014) and **(B)** the short-rain season (October, 2014–January, 2015).

DATIC site received more rainfall (average, 5.30 mm) than MUARIK (average, 2.03 mm) during the long-rain season. However, during the short-rain season, MUARIK received slightly more rainfall (average, 2.74 mm) than DATIC (average, 2.44 mm). The rainfall was unevenly distributed across sites and seasons. In the short-rain season, there was almost no rain for ~6 weeks in both sites. The rain distribution and intensity at DATIC was better than MUARIK during the long-rain season. At MUARIK the intensity was higher at the beginning of the short-rain season, although the rains ceased too early (Figures 2A,B).

### Sweet potato AMF root colonization intensity

**Figure 3:**
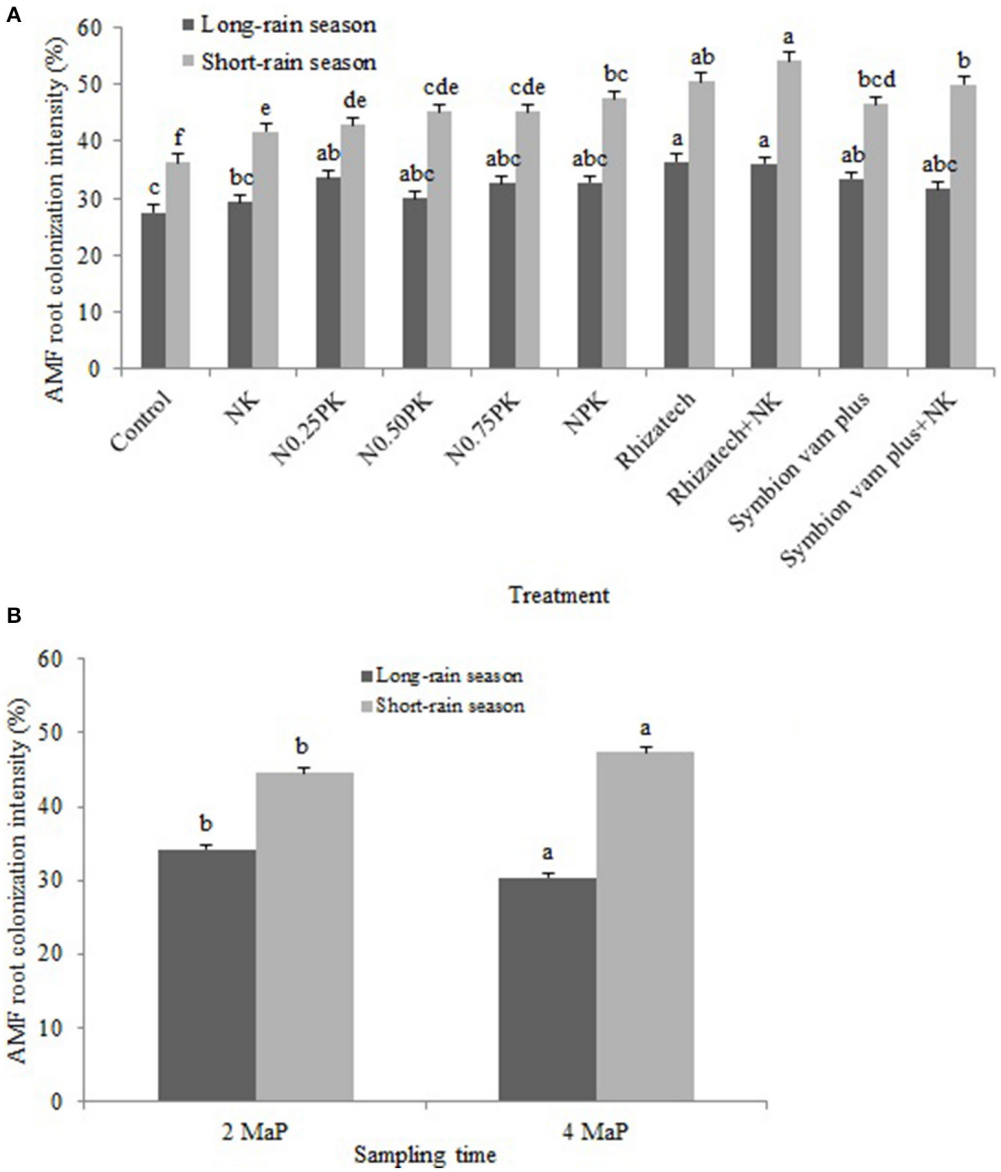
**(A)** Arbuscular mycorrhizal fungi root colonization intensity as influenced by the interaction of treatment and season; **(B)** Arbuscular mycorrhizal fungi root colonization intensity as influenced by the interaction of sampling time and season.

Root colonization intensity differed by the interaction of treatments and seasons (*p* = 0.0007), treatment composition (*p* < 0.0001) and seasonal variation (*p* < 0.0001; Figure 3A), and the sampling date and season (*p* < 0.0001) as shown in Figure 3B.

The intensity of AMF root colonization was greater in the short-rain season than in the long-rain season in both sites with a range of 31.0–55.3% and 24.0–42.7%, respectively (Table 3).

**Table 3:** **Sweet potato arbuscular mycorrhizal fungi root colonization intensity across treatments**.

	**DATIC (Ferralsol)**		**MUARIK (Rhodic Nitisol)**	
**Treatment**	**Colonization2 (%)**	**Colonization4 (%)**	**Colonization2 (%)**	**Colonization4 (%)**
**LONG-RAIN SEASON**				
Control	30.6	24.0	30.3^c^	25.3
NK	31.3	28.6	30.3^c^	26.7
N0.25PK	34.9	36.2	35.0^ab^	28.0
N0.50PK	29.5	30.7	31.0^bc^	28.3
N0.75PK	32.3	29.7	35.3^ab^	33.0
NPK	33.6	32.7	35.3^ab^	29.2
Rhizatech	33.6	32.6	42.7a	37.0
Rhizatech+NK	37.5	31.8	41.1a	33.9
Symbion vam plus	33.8	30.3	38.9^ab^	30.5
Symbion vam plus+NK	32.3	29.6	34.5^ab^	30.1
*P*-value	0.3635	0.4261	0.0345	0.3188
**Treatment Contrasts**	***F*****-value (*****p*****-value)**			
1 vs. 2,3,4,5,6	0.44 (Ns)	5.04 (Ns)	0.94 (Ns)	1.30 (Ns)
1 vs. 7,8,9,10	2.25 (Ns)	4.22 (Ns)	7.70 (0.0099^*^)	4.68 (Ns)
2 vs. 3,4,5,6	0.29 (Ns)	1.14 (Ns)	1.38 (Ns)	0.78 (Ns)
2 vs. 7,8,9,10	1.64 (Ns)	0.50 (Ns)	7.63 (0.0102^*^)	3.06 (Ns)
3,4,5,6 vs. 7,8	2.69 (Ns)	0.00 (Ns)	6.46 (0.0171^*^)	4.17 (Ns)
3,4,5,6 vs. 9,10	0.08 (Ns)	0.79 (Ns)	2.43 (Ns)	0.04 (Ns)
7,8 vs. 9,10	1.40 (Ns)	0.54 (Ns)	0.72 (Ns)	2.54 (Ns)
7 vs. 8	1.70 (Ns)	0.04 (Ns)	0.08 (Ns)	0.38 (Ns)
9 vs. 10	0.22 (Ns)	0.02 (Ns)	3.00 (Ns)	0.01 (Ns)
SE	2.11	3.08	2.93	3.10
**SHORT-RAIN SEASON**				
Control	31.0^e^	41.3^c^	33.7^d^	39.3^e^
NK	40.7^cde^	43.6^bc^	38.7^cd^	43.7^de^
N0.25PK	39.7^de^	44.0^bc^	43.3^bc^	44.3^cde^
N0.50PK	43.3^bcd^	46.2^abc^	44.7^bc^	46.3^bcd^
N0.75PK	43.3^bcd^	46.7^abc^	44.3^bc^	46.3^bcd^
NPK	45.3^bcd^	47.2^abc^	47.0^abc^	50.3^ab^
Rhizatech	51.3^ab^	50.9^a^	47.3^abc^	52.8^a^
Rhizatech+NK	55.3^a^	52.3^a^	55.3^a^	54.0^a^
Symbion vam plus	46.0^abcd^	47.0^abc^	43.7^bc^	49.3^abc^
Symbion vam plus+NK	50.0^abc^	49.3^ab^	48.2^ab^	52.3^a^
*P*-value	<0.0001	0.0321	<0.0001	<0.0001
**Treatment Contrasts**	***F*****-value (*****p*****-value)**			
1 vs. 2,3,4,5,6	29.69 (<0.0001^*^)	3.58 (Ns)	23.76 (0.0001^*^)	34.74 (<0.0001^*^)
1 vs. 7,8,9,10	83.85 (<0.0001^*^)	14.22 (0.0014^*^)	51.72 (<0.0001^*^)	115.37 (<0.0001^*^)
2 vs. 3,4,5,6	1.10 (Ns)	1.10 (Ns)	8.79 (0.0083^*^)	7.09 (0.0158^*^)
2 vs. 7,8,9,10	21.68 (0.0002^*^)	7.59 (0.0130^*^)	22.92 (0.0001^*^)	50.36 (<0.0001^*^)
3,4,5,6 vs. 7,8	39.21 (<0.0001^*^)	10.17 (0.0051^*^)	16.28 (0.0008^*^)	50.45 (<0.0001^*^)
3,4,5,6 vs. 9,10	9.34 (0.0068^*^)	1.49 (Ns)	0.45 (Ns)	18.86 (0.0004^*^)
7,8 vs. 9,10	7.71 (0.0125^*^)	2.90 (Ns)	8.48 (0.0093^*^)	5.71 (0.0280^*^)
7 vs. 8	2.17 (Ns)	0.24 (Ns)	9.25 (0.0070^*^)	0.69 (Ns)
9 vs. 10	2.17 (Ns)	0.66 (Ns)	2.93 (Ns)	3.98 (Ns)
SE	1.92	2.03	1.86	1.06

*Colonization2 and Colonization4 = AMF root colonization intensity at 2 and 4 MaP, respectively. 1 = Control, 2 = NK, 3 = N0.25PK, 4 = N0.50PK, 5 = N0.75PK, 6 = NPK, 7 = Rhizatech, 8 = Rhizatech+NK, 9 = Symbion vam plus, 10 = Symbion vam plus+NK. Means were separated using Tukey HSD test (p ≤ 0.05). Mean values in the same column followed by the same letters are not significantly different (p > 0.05). Treatment contrasts: 1 vs. 2,3,4,5,6 = Control vs. NPK treatments; 1 vs. 7,8,9,10 = Control vs. Biofertilizer treatments; 2 vs. 3,4,5,6 = NK vs. NPK; 2 vs. 7,8,9,10 = NK vs. Biofertilizer treatments; 3,4,5,6 vs.7,8 = NPK vs. Rhizatech treatments; 3,4,5,6 vs.7,8 = NPK vs. Symbion vam plus treatments; 7,8 vs. 9,10 = Rhizatech treatments vs. Symbion vam plus treatments; 7 vs. 8 = Rhizatech vs. Rhizatech+NK and 9 vs. 10 = Symbion vam plus vs. Symbion vam plus+NK*.

* *significantly different at p ≤ 0.05; Ns = not significant at p > 0.05; SE = standard error. Treatments with the largest mean values contributed to significant differences in the different contrast groups*.

In the Ferralsol, contrast analysis showed that applying inorganic fertilizers or biofertilizers with and without N and K significantly increased colonization intensity compared to the control and NK in the short-rain season but not in the long-rain season. Applying Rhizatech or Symbion vam plus with or without N and K significantly increased colonization compared to the NPK treatments at 2 and 4 MaP. Effect of Rhizatech with or without N and K was significantly greater than Symbion vam plus with or without N and K at 2 MaP. Biofertilizers applied singly or in combination with N and K significantly increased colonization intensity compared to the control. Rhizatech with or without N and K had significantly more colonization compared to the NPK treatments at 4 MaP (Table 3).

In the Rhodic Nitisol, applying biofertilizers singly or with N and K significantly increased colonization intensity compared to the control. Rhizatech with or without N and K had significantly more colonization compared to the NPK treatments at 2 MaP in the long-rain season. Biofertilizers applied singly or in combination with N and K significantly increased colonization intensity compared to NK at 2 MaP in the long-rain season. In the short-rain season, applying inorganic fertilizer treatments and biofertilizers with or without N and K significantly had more colonization compared to the control. In the same season, NPK treatments and biofertilizers with or without N and K significantly increased colonization compared to NK while Rhizatech, with or without N and K, significantly increased colonization compared to the NPK treatments at 2 and 4 MaP. Symbion vam plus with or without N and K and Rhizatech combined with N and K significantly improved colonization compared to Rhizatech alone at 2 MaP. At 4 MaP, applying inorganic fertilizer treatments or biofertilizers with or without N and K significantly increased colonization intensity over the control. The NPK treatments significantly improved colonization intensity compared to NK while Rhizatech with or without N and K and Symbion vam plus with or without N and K significantly performed better compared to the NPK treatments. Rhizatech with or without N and K significantly performed better compared to Symbion vam plus with or without N and K in improving colonization intensity (Table 3).

### Nutrient recovery

**Figure 4:**
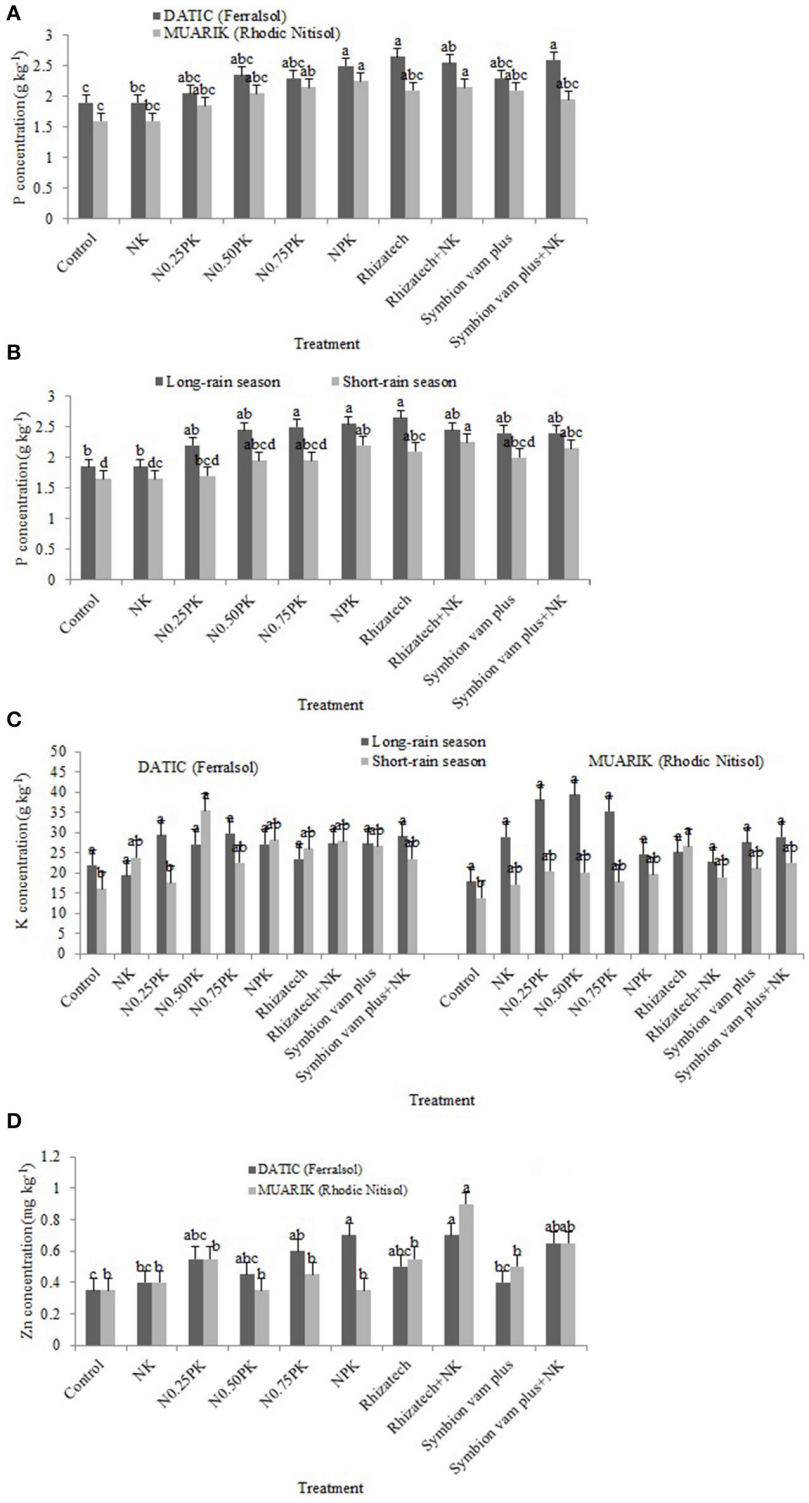
**Phosphorus concentration (A)** influenced by treatment and soil type; **(B)** influenced by treatment and season; **(C)** potassium concentration as influenced by the interaction of treatment, soil type and season; **(D)** zinc concentration as influenced by the interaction of treatment and soil type.

Generally, P concentration was influenced by treatment composition (*p* < 0.0001), soil type (*p* < 0.0001) and variations in the seasons (*p* < 0.0001; Figures 4A,B). Potassium concentration was influenced by the interaction of treatment, soil type and season (*p* = 0.0403), soil type (*p* = 0.0042) and differences in seasons (*p* = 0.0004; Figure 4C). Zinc concentration was significantly influenced by the interaction of treatment and soil type (*p* = 0.0191), and treatment composition (*p* < 0.0001; Figure 4D).

Table 4 shows the means of nutrient concentration and treatment contrast analysis for each site (soil type), respectively, and for both seasons. Treatments variably affected the concentration of macronutrients (P and K) and micronutrient (Zn) in sweet potato vines in the two soil types over the seasons (Table 4).

**Table 4:** **Sweet potato vines nutrient concentration across treatments**.

	**DATIC (Ferralsol)**			**MUARIK (Rhodic Nitisol)**		
**Treatment**	**P (g kg^−1^)**	**K (g kg^−1^)**	**Zn (g kg^−1^)**	**P (g kg^−1^)**	**K (g kg^−1^)**	**Zn (g kg^−1^)**
**LONG-RAIN SEASON**						
Control	2.00	21.9	0.035^bc^	1.70^b^	17.8	0.041^b^
NK	2.00	19.4	0.038^bc^	1.73^b^	28.9	0.028^b^
N0.25PK	2.33	29.3	0.046^bc^	2.13^ab^	38.2	0.029^b^
N0.50PK	2.58	27.1	0.054^ab^	2.30^ab^	39.3	0.034^b^
N0.75PK	2.60	29.8	0.068^a^	2.38^a^	35.3	0.037^b^
NPK	2.75	27.1	0.067^a^	2.30^ab^	24.6	0.029^b^
Rhizatech	2.93	23.4	0.055^ab^	2.38^a^	25.2	0.042^b^
Rhizatech+NK	2.48	27.3	0.069^a^	2.35^a^	22.7	0.114^a^
Symbion vam plus	2.45	27.3	0.031^c^	2.30^ab^	27.5	0.042^b^
Symbion vam plus+NK	2.75	29.1	0.056^ab^	1.98^ab^	28.9	0.068^ab^
*P*-value	0.0595	0.0689	0.0008	0.0456	0.1890	<0.0001
**Treatment Contrasts**	***F*****-value (*****p*****-value)**					
1 vs. 2,3,4,5,6	3.84 (Ns)	3.13 (Ns)	7.32 (0.0116^*^)	5.24 (0.0301^*^)	6.32 (Ns)	0.73 (Ns)
1 vs. 7,8,9,10	7.69 (Ns)	3.32 (Ns)	5.70 (0.0242^*^)	7.33 (0.0116^*^)	1.73 (Ns)	4.54 (0.0425^*^)
2 vs. 3,4,5,6	5.76 (Ns)	1.14 (Ns)	8.20 (0.0080^*^)	8.79 (0.0063^*^)	0.76 (Ns)	0.16 (Ns)
2 vs. 7,8,9,10	7.69 (0.0099^*^)	7.62 (0.0102^*^)	4.11 (0.0526^*^)	8.04 (0.0086^*^)	0.20 (Ns)	10.81 (0.0028^*^)-
3,4,5,6 vs. 7,8	0.57 (Ns)	2.02 (Ns)	0.30 (Ns)	0.34 (Ns)	4.61 (Ns)	25.09 (<0.0001^*^)
3,4,5,6 vs. 9,10	0.04 (Ns)	0.00 (Ns)	7.31 (0.0117^*^)	0.84 (Ns)	1.61 (Ns)	6.01 (0.0210^*^)
7,8 vs. 9,10	0.23 (Ns)	1.37 (Ns)	7.91 (0.0091^*^)	1.68 (Ns)	0.58 (Ns)	4.91 (0.0354^*^)
7 vs. 8	2.30 (Ns)	1.30 (Ns)	2.50 (Ns)	0.01 (Ns)	0.10 (Ns)	23.36 (<0.0001^*^)
9 vs. 10	1.02 (Ns)	0.26 (Ns)	7.91 (0.0091^*^)	1.75 (Ns)	0.03 (Ns)	3.01 (Ns)
SE	0.23	0.24	0.006	0.02	0.56	0.011
**SHORT-RAIN SEASON**						
Control	1.73^b^	16.1^b^	0.025	1.46^b^	13.7^b^	0.034
NK	1.80^ab^	23.8^ab^	0.039	1.53b	17.1^ab^	0.050
N0.25PK	1.83^ab^	17.6^b^	0.056	1.67^ab^	20.4^ab^	0.079
N0.50PK	2.07^ab^	35.4^a^	0.043	1.77^ab^	20.2^ab^	0.043
N0.75PK	2.03^ab^	22.5^ab^	0.055	1.87^ab^	17.8^ab^	0.055
NPK	2.23^ab^	28.2^ab^	0.073	2.23^a^	19.6^ab^	0.037
Rhizatech	2.37^ab^	26.1^ab^	0.050	1.83^ab^	26.7^a^	0.066
Rhizatech+NK	2.57^a^	27.9^ab^	0.071	1.93^ab^	18.9^ab^	0.072
Symbion vam plus	2.10^ab^	26.6^ab^	0.051	1.87^ab^	21.2^ab^	0.056
Symbion vam plus+NK	2.37^ab^	23.5^ab^	0.069	1.87^ab^	22.5^ab^	0.064
*P*-value	0.0152	0.0396	0.1570	0.0482	0.0385	0.4865
**Treatment Contrasts**	***F*****-value (*****p*****-value)**					
1 vs. 2,3,4,5,6	2.46 (0.0333^*^)	6.35 (0.0363^*^)	4.75 (Ns)	5.31 (0.0333^*^)	5.12 (0.0363^*^)	1.32 (Ns)
1 vs. 7,8,9,10	13.28 (0.0159^*^)	6.84 (0.0020^*^)	7.24 (Ns)	7.08 (0.0159^*^)	12.99 (0.0020^*^)	3.36 (Ns)
2 vs. 3,4,5,6	2.04 (0.0350^*^)	0.31 (Ns)	1.95 (Ns)	5.20 (0.0350^*^)	1.05 (Ns)	0.04 (Ns)
2 vs. 7,8,9,10	10.56 (0.0044^*^)	0.35 (Ns)	2.80 (Ns)	4.95 (0.0391^*^)	4.87 (0.0406^*^)	0.74 (Ns)
3,4,5,6 vs. 7,8	10.51 (Ns)	0.14 (Ns)	0.15 (Ns)	0.00 (Ns)	3.26 (Ns)	1.48 (Ns)
3,4,5,6 vs. 9,10	2.14 (Ns)	0.08 (Ns)	0.10 (Ns)	0.02 (Ns)	1.56 (Ns)	0.26 (Ns)
7,8 vs. 9,10	2.38 (Ns)	0.32 (Ns)	0.00 (Ns)	0.01 (Ns)	0.23 (Ns)	0.37 (Ns)
7 vs. 8	0.87 (Ns)	0.14 (0.0167^*^)	1.73 (Ns)	0.27 (Ns)	6.95 (0.0167^*^)	0.07 (Ns)
9 vs. 10	1.55 (Ns)	0.41 (Ns)	1.13 (Ns)	0.00 (Ns)	0.18 (Ns)	0.13 (Ns)
SE	0.02	0.34	0.012	0.01	0.21	0.015

*P = phosphorus; K = potassium; Zn = zinc; 1 = Control, 2 = NK, 3 = N0.25PK, 4 = N0.50PK, 5 = N0.75PK, 6 = NPK, 7 = Rhizatech, 8 = Rhizatech+NK, 9 = Symbion vam plus, 10 = Symbion vam plus+NK. Means were separated using Tukey HSD test (p ≤ 0.05). Mean values in the same column followed by the same letters are not significantly different (p > 0.05). Treatment contrasts: 1 vs. 2,3,4,5,6 = Control vs. NPK treatments; 1 vs. 7,8,9,10 = Control vs. Biofertilizer treatments; 2 vs. 3,4,5,6 = NK vs. NPK; 2 vs. 7,8,9,10 = NK vs. Biofertilizer treatments; 3,4,5,6 vs.7,8 = NPK vs. Rhizatech treatments; 3,4,5,6 vs.7,8 = NPK vs. Symbion vam plus treatments; 7,8 vs. 9,10 = Rhizatech treatments vs. Symbion vam plus treatments; 7 vs. 8 = Rhizatech vs. Rhizatech+NK and 9 vs. 10 = Symbion vam plus vs. Symbion vam plus+NK*.

* *significantly different at p ≤ 0.05; Ns = not significant at p > 0.05; SE = standard error. Treatments with the largest mean values contributed to significant differences in the different contrast groups*.

In the Ferralsol, the contrast analysis showed that biofertilizers with or without N and K significantly increased P concentration when compared to the control in the short-rain season and NK in the short and long-rain season. In the same season, Rhizatech with or without N and K significantly outperformed NPK treatments (Table 4). For the two seasons in the Rhodic Nitisol, inorganic fertilizers significantly increased P concentration compared to the control. However, NPK treatments significantly increased P concentration compared to the NK treatment in the two seasons. Biofertilizers with or without N and K significantly increased P concentration when compared to the control and NK in the two seasons.

The effect of biofertilizers with or without N and K and inorganic fertilizer treatments on K concentration was significantly greater compared to the control in the short-rain season in the Ferralsol. Biofertilizers with or without N and K outperformed NK in the long-rain season in the Ferralsol. In the Rhodic Nitisol, applying biofertilizers with or without N and K significantly increased K concentration beyond the control and the NK in the short-rain season. Rhizatech+NK significantly increased K concentration compared to Rhizatech applied singly while the inorganic fertilizer treatments significantly outperformed the control in the short-rain season (Table 4).

Significant differences in nutrient concentrations were observed where biofertilizers with or without N and K, and inorganic fertilizers were applied compared to the control and NK during the long-rain season in both soils. In the same season, biofertilizers applied singly or supplemented with N and K significantly increased Zn concentration beyond the inorganic fertilizer treatments in both soils. However, NPK treatments significantly outperformed NK treatment while Symbion vam plus supplemented with N and K significantly outperformed Symbion vam plus alone in the Ferralsol during the long-rain season. In the Rhodic Nitisol, Rhizatech with or without N and K significantly increased Zn concentration compared to Symbion vam plus with or without N and K in the long-rain season (Table 4).

### Biomass accumulation and tuber yield

**Figure 5:**
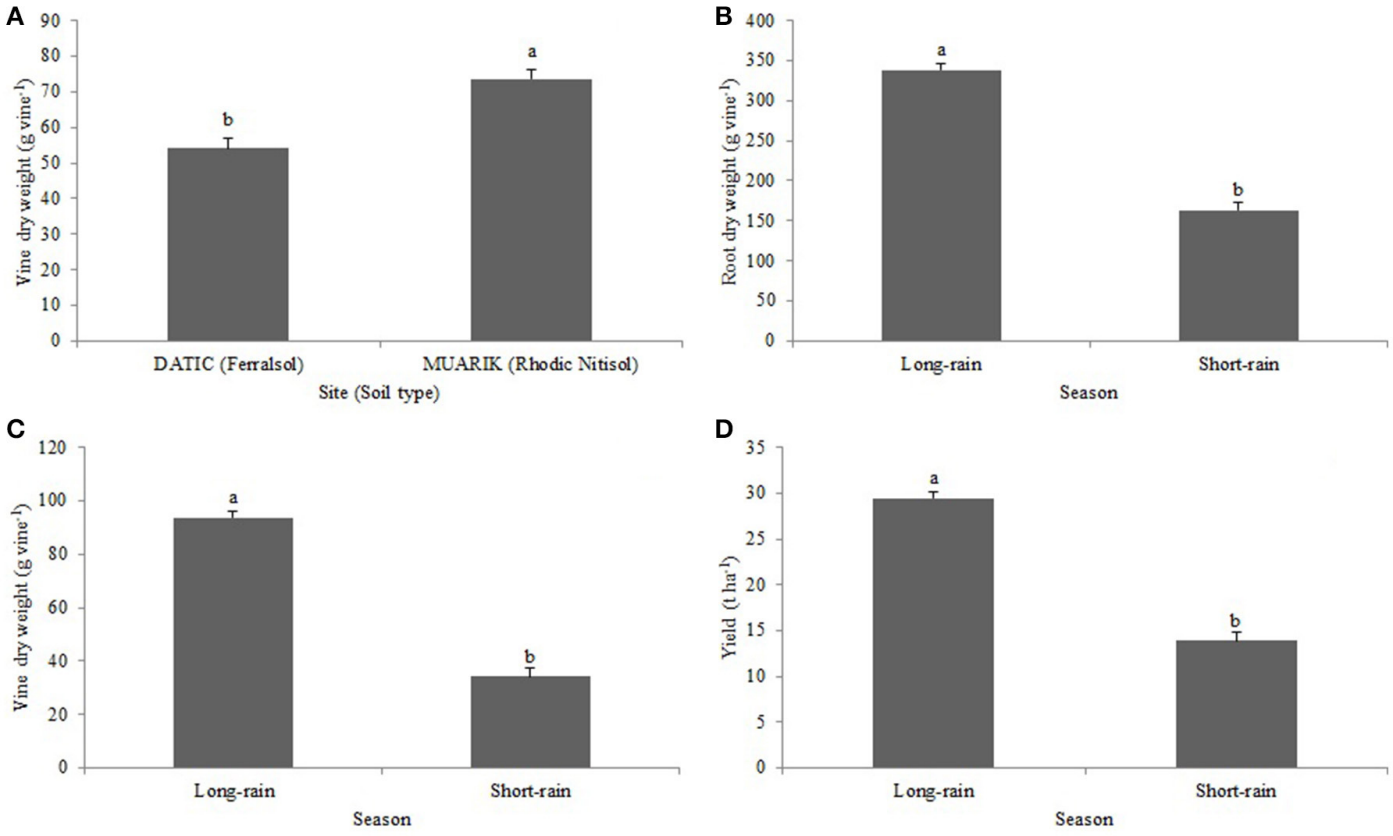
**Effect of (A)** soil type on vine dry weight **(B)** season on vine dry weight; **(C)** season on root dry weight; **(D)** season on yield.

Vine dry weight was significantly influenced by soil type (*p* < 0.0001; Figure 5A) and season (*p* < 0.0001; Figure 5B). The root dry weight was significantly (*p* < 0.0001) influenced by seasonal changes (Figure 5C). The tuber yield was significantly (*p* < 0.0001) affected by variations in seasons (Figure 5D).

Insignificant effects of treatments were observed on growth and tuber yield except for yield in the Ferralsol during the short-rain season (Table 5). Contrast analysis indicated that the inorganic fertilizer treatments and the biofertilizers with or without N and K significantly increased yield as compared to the control while the effect of NPK treatments on yield was significantly greater compared to that of NK.

**Table 5:** **Growth of sweet potato vines and roots, and yield at 4 months at planting**.

	**DATIC (Ferralsol)**			**MUARIK (Rhodic Nitisol)**		
**Treatment**	**^a^VDW (g)**	**^b^RDW (g)**	**Tuber yield (t ha−1)**	**^a^VDW (g)**	**^b^RDW (g)**	**Tuber yield (t ha−1)**
**LONG-RAIN SEASON**						
Control	63.3	329.3	27.6	101.2	277.7	25.8
NK	91.4	400.3	34.5	101.4	331.1	28.3
N0.25PK	87.2	389.2	34.6	98.4	324.5	29.8
N0.50PK	76.5	381.8	30.5	102.4	291.9	27.6
N0.75PK	75.8	321.5	33.5	125.2	388.6	32.9
NPK	106.2	461.3	34.5	117.2	329.4	28.3
Rhizatech	65.8	346.4	34.3	97.5	252.4	21.4
Rhizatech+NK	106.0	352.5	30.5	112.3	316.5	27.5
Symbion vam plus	60.8	292.4	23.8	84.9	346.3	28.8
Symbion vam plus+NK	80.2	324.3	27.8	119.6	300.6	26.3
*P*-value	0.23	0.46	0.56	0.50	0.30	0.61
**Treatment Contrasts**	***F*****-value (*****p*****-value)**					
1 vs. 2,3,4,5,6	2.54 (Ns)	1.31 (Ns)	1.79 (Ns)	0.34 (Ns)	0.00 (Ns)	0.97 (Ns)
1 vs. 7,8,9,10	0.93 (Ns)	0.00 (Ns)	0.12 (Ns)	0.02 (Ns)	0.87 (Ns)	0.00 (Ns)
2 vs. 3,4,5,6	0.10 (Ns)	0.05 (Ns)	0.07 (Ns)	0.49 (Ns)	0.02 (Ns)	0.15 (Ns)
2 vs. 7,8,9,10	0.73 (Ns)	1.70 (Ns)	1.41 (Ns)	0.01 (Ns)	0.63 (Ns)	0.37 (Ns)
3,4,5,6 vs. 7,8	0.00 (Ns)	0.84 (Ns)	0.06 (Ns)	0.45 (Ns)	3.88 (Ns)	3.33 (Ns)
3,4,5,6 vs. 9,10	1.78 (Ns)	3.57 (Ns)	4.50 (Ns)	0.69 (Ns)	0.20 (Ns)	0.55 (Ns)
7,8 vs. 9,10	1.25 (Ns)	0.71 (Ns)	2.65 (Ns)	0.02 (Ns)	1.74 (Ns)	0.88 (Ns)
7 vs. 8	4.25 (Ns)	0.01 (Ns)	0.45 (Ns)	0.86 (Ns)	2.50 (Ns)	1.71 (Ns)
9 vs. 10	1.00 (Ns)	0.21 (Ns)	0.49 (Ns)	3.75 (Ns)	0.90 (Ns)	0.31 (Ns)
SE	13.77	49.01	4.05	12.66	34.06	3.29
**SHORT-RAIN SEASON**						
Control	29.1	89.6	7.6^b^	39.1	159.4	13.6
NK	17.6	105.3	9.6^a^^b^	43.2	166.7	13.8
N0.25PK	34.2	185.8	14.9^a^	47.0	197.8	17.7
N0.50PK	24.1	171.4	13.6^a^^b^	36.6	223.3	18.4
N0.75PK	26.3	173.1	12.9^a^^b^	47.6	182.9	15.8
NPK	31.9	140.9	12.7^a^^b^	40.4	201.9	15.7
Rhizatech	23.8	135.2	11.3^a^^b^	26.4	116.7	12.8
Rhizatech+NK	29.5	145.4	12.8^a^^b^	44.9	153.0	13.2
Symbion vam plus	19.6	112.0	9.9^a^^b^	39.9	205.0	18.0
Symbion vam plus+NK	33.6	156.1	13.6^a^^b^	48.2	237.5	20.1
*P*-value	0.26	0.11	0.02	0.73	0.18	0.28
**Treatment Contrasts**	***F*****-value (*****p*****-value)**					
1 vs. 2,3,4,5,6	0.19 (Ns)	6.93 (Ns)	13.09 (0.0020^*^)	0.19 (Ns)	1.28 (Ns)	1.27 (Ns)
1 vs. 7,8,9,10	0.21 (Ns)	3.48 (Ns)	8.90 (0.0080^*^)	0.01 (Ns)	0.35 (Ns)	1.01 (Ns)
2 vs. 3,4,5,6	4.59 (Ns)	6.03 (Ns)	7.36 (0.0143^*^)	0.00 (Ns)	1.20 (Ns)	1.60 (Ns)
2 vs. 7,8,9,10	2.83 (Ns)	1.57 (Ns)	2.59 (Ns)	0.14 (Ns)	0.13 (Ns)	0.84 (Ns)
3,4,5,6 vs. 7,8	0.34 (Ns)	1.94 (Ns)	1.63 (Ns)	1.09 (Ns)	7.35 (Ns)	4.18 (Ns)
3,4,5,6 vs. 9,10	0.36 (Ns)	2.93 (Ns)	2.48 (Ns)	0.03 (Ns)	0.65 (Ns)	1.29 (Ns)
7,8 vs. 9,10	0.00 (Ns)	0.08 (Ns)	0.07 (Ns)	1.10 (Ns)	9.27 (Ns)	0.02 (Ns)
7 vs. 8	0.70 (Ns)	0.10 (Ns)	0.62 (Ns)	2.63 (Ns)	0.82 (Ns)	0.07 (Ns)
9 vs. 10	4.17 (Ns)	1.88 (Ns)	3.98 (Ns)	0.53 (Ns)	0.65 (Ns)	0.46 (Ns)
SE	4.83	22.78	1.30	8.07	28.39	2.20

a *VDW = vine dry weight*,

b *RDW = root dry weight; 1 = Control, 2 = NK, 3 = N0.25PK, 4 = N0.50PK, 5 = N0.75PK, 6 = NPK, 7 = Rhizatech, 8 = Rhizatech+NK, 9 = Symbion vam plus, 10 = Symbion vam plus+NK. Means were separated using Tukey HSD test (p ≤ 0.05). Mean values in the same column followed by the same letters are not significantly different (p > 0.05). Treatment contrasts: 1 vs. 2,3,4,5,6 = Control vs. NPK treatments; 1 vs. 7,8,9,10 = Control vs. Biofertilizer treatments; 2 vs. 3,4,5,6 = NK vs. NPK; 2 vs. 7,8,9,10 = NK vs. Biofertilizer treatments; 3,4,5,6 vs.7,8 = NPK vs. Rhizatech treatments; 3,4,5,6 vs.7,8 = NPK vs. Symbion vam plus treatments; 7,8 vs. 9,10 = Rhizatech treatments vs. Symbion vam plus treatments; 7 vs. 8 = Rhizatech vs. Rhizatech+NK and 9 vs. 10 = Symbion vam plus vs. Symbion vam plus+NK*.

* *significantly different at p ≤ 0.05; Ns = not significant at p > 0.05; SE = standard error. Treatments with the largest mean values contributed to significant differences in the different contrast groups*.

### Linear regression analysis

A simple linear regression analysis showed some significant relationship between AMF root colonization and nutrient uptake, but the coefficients of determination (*r*^2^) were relatively low. Sweet potato P concentration was positively correlated with AMF root colonization at 4 MaP in the Ferralsol (*r*^2^ = 0.233; *p* = 0.007) and Rhodic Nitisol (*r*^2^ = 0.126; *p* = 0.054) during the short-rain season. Sweet potato vine Zn concentration was positively correlated (*r*^2^ = 0.3834; *p* = 00003) with AMF root colonization in the Ferralsol at 4 MaP (*r*^2^ = 0.3900; *p* < 0.001) and 4 MaP (*r*^2^ = 0.3834; *p* < 0.001). According to this analysis, AMF root colonization never correlated with the uptake of K and sweet potato biomass accumulation and tuber yield. However, given the low coefficients of determination, AMF root colonization only would not be adequate enough to predict P and Zn uptake in sweet potato, but the biofertilizers significantly contributed to the nutrient uptake. The tuber yield was positively influenced (*r*^2^ = 0.0809; *p* = 0.0105) by vine growth during the long-rain season in the Ferralsol, and during the long-rain season (*r*^2^ = 0.3021; *p* = 0.0002) and the short-rain season (*r*^2^ = 0.2917; *p* = 0.0021) in the Rhodic Nitisol, although the coefficients of determination (*r*^2^) were relatively low (Table 6).

**Table 6:** **Linear regression of arbuscular mycorrhizal fungi root colonization with phosphorus, potassium, and zinc uptake, vine and root dry weight, and tuber yield**.

		**Dependent variable**					
**Independent variable**	**Site/Season**	**P**	**K**	**Zn**	**aVDW**	**bRDW**	**Tuber yield**
Colonization4	*DATIC*						
	*Long-rain*						
	Estimate	0.001	0.020	−0.00003	0.70	2.54	0.32
	SE	0.001	0.014	0.00046	0.72	2.66	0.22
	*T*-value	1.180	1.540	−0.07000	0.97	0.95	1.47
	*P*-value	0.246	0.159	0.94280	0.34	0.35	0.15
	*R*^2^	0.035	0.051	0.00014	0.02	0.02	0.54
^a^VDW	Estimate						0.09
	SE						0.03
	*T*-value						2.62
	*P*-value						0.01
	*R*^2^						0.08
Colonization4	*DATIC*						
	*Short-rain*						
	Estimate	0.004	0.009	0.0031	0.33	1.26	0.16
	SE	0.001	0.031	0.0008	0.39	2.06	0.15
	*T*-value	2.920	0.290	4.1700	0.85	0.61	1.10
	*P*-value	0.007	0.770	0.0003	0.40	0.55	0.28
	*R*^2^	0.233	0.003	0.3834	0.03	0.01	0.04
^a^VDW	Estimate						0.10
	SE						0.07
	*T*-value						1.43
	*P*-value						0.17
	*R*^2^						0.07
Colonization4	*MUARIK*						
	Long-rain						
	Estimate	0.001	0.005	0.0001	−0.39	−2.23	−0.25
	SE	0.001	0.028	0.0007	0.64	2.23	0.17
	*T*-value	1.010	0.170	0.1700	−0.62	−1.00	−1.47
	*P*-value	0.321	0.867	0.8648	0.54	0.32	0.15
	*R*^2^	0.026	0.001	0.0008	0.01	0.03	0.05
^a^VDW	Estimate						0.15
	SE						0.04
	*T*-value						4.06
	*P*-value						0.0002
	R2						0.30
Colonization4	*MUARIK*						
	Short-rain						
	Estimate	0.002	0.030	0.0007	0.51	1.37	0.15
	SE	0.001	0.019	0.0009	0.53	2.14	0.16
	*T*-value	2.010	1.540	0.7600	0.95	0.64	0.96
	*P*-value	0.054	0.134	0.4538	0.35	0.53	0.35
	*R*^2^	0.126	0.079	0.0202	0.03	0.01	0.03
^a^VDW	Estimate						0.16
	SE						0.05
	*T*-value						3.40
	*P*-value						0.0021
	*R*^2^						0.29

*Colonization4, AMF root colonization intensity at 4 MaP; P, phosphorus; K, potassium; Zn, zinc; aVDW, vine dry weight, bRDW, root dry weight*.

## Discussion

The sweet potato root colonization, nutrient concentration and, growth and yield were variably affected by treatment composition, soil conditions, seasonal variations, and the interactions of these factors.

### Fertilization effectiveness in sweet potato production

In this study, it was hypothesized that AMF increases bioavailability and uptake of P required by the sweet potato crop hence the comparison of biofertilizer + NK with NPK. However, the P mobilized by AMF in these low P soils was generally lower than what was supplied by the NPK treatments. Response to AMF is expected to be better in slightly acidic soils (Fattah, 2013), like the ones used in this study, by improved solubilization of P or extended rhizosphere by fungal hyphae (Lambers et al., 2008; Parewa et al., 2010) increasing surface area for nutrient acquisition. Generally, the effect of biofertilizer on root colonization, nutrient uptake and, growth and yield was not always significant which is possibly due to the shorter period (4 months) the crop was maintained in the field or due to low available P in the soil. The low response to mycorrhizal treatments in growth of annual crops could be attributed to the fact that colonization commences after hyphal formation and subsequent colonization of the root. This depends on the state of propagules (spores, hyphae) which may take long period to germinate and infect the plant. Spores persist longer in the soil but they are slow to colonize host plants as compared to hyphae and root fragments (Marin, 2006; Mukhongo et al., 2016). Kavoo-Mwangi et al. (2013) reported lack of immediate expression of Rhizatech (*G. mosseae, G. intraradices, G. etunicatum*, and *G. aggregatum*) in growth of tissue culture banana plantlets for 22 weeks under nursery conditions, but they gave significant results when the inoculated plantlets were established under field conditions for 7 months. The less significant results could also be due to the low spore densities in both experimental soils (4–5 spores g^−1^) and biofertilizers (2–4 spores g^−1^) as compared to high density (50 propagules g^−1^) used by Kavoo-Mwangi et al. (2013). However, it is possible that the occurrence of both indigenous and introduced AMF in plots that received biofertilizer treatments boosted the AMF spores in soil, and hence the significant colonization over the rest of the treatments. Although it is unknown if spore abundance increased during this experiment as this was measured only before planting. Increased abundance of AMF propagules is important to achieve rapid colonization (Verbruggen et al., 2012). This was also supported by the effect of Rhizatech (4 spores g^−1^ of product) with or without N and K which was in most cases greater than for Symbion vam plus (2 spores g^−1^ of product) with or without N and K. Combining biofertilizers with N and K consistently performed better than biofertilizers alone which demonstrated the importance of starter dose of nutrients in low nutrient soils like the Ferralsol and the Rhodic Nitisol used in the present study. However, due to the lack of data on AMF changes in spore abundance, hyphal biomass and diversity (in spores and in plant roots) after biofertilizers were applied, and lack of a biofertilizer + NPK treatment in this study, we were unable to separate native verse biofertilizer AMF effects on sweet potato root colonization, nutrient uptake, biomass accumulation, and tuber yield.

Phosphorus and zinc were analyzed in the sweet potato vines because AMF has frequently been reported to increase their availability and concentration, while potassium is required in large quantities by sweet potato. The significant effect of biofertilizers with or without N and K on increasing P, K, and Zn concentration was attributed to the functioning of AMF and *B. megaterium* present in the biofertilizers. Root colonization by AMF improves nutrient uptake per unit of root length due to the enhancement of root surface area by hyphal growth and providing an extra route for uptake as mycorrhizal pathway (Smith et al., 2003) but this depends on the AMF species colonizing the plant roots. Efficiency of AMF species is influenced differently by their development and activity of the external hyphae, hyphal transport rates, and solute interchange at the arbuscule-host root cell interface (Marschner, 1995; Hajiboland et al., 2009). Plant growth promoting bacterium *B. megaterium* improves root growth and development (Mia et al., 2002; Kavoo-Mwangi et al., 2013) hence increased nutrient uptake. During drought conditions in the low fertility Ferralsol soil, AMF root colonization increased with water stress (Auge, 2001) therefore, increasing P and K uptake through solubilization and extended rhizosphere due to fungal hyphae. Mycorrhizae are known to help plants acquire nutrients that are fixed in the soil such as P and also significantly improve plant nutrition under low fertility soil conditions (Lambers et al., 2008, 2011). The major ways of increasing uptake of nutrients include increase of acquisition area by root growth or involvement of mycorrhizae and root exudation of low molecular weight organic acids (LMWOAs) or phytosiderophores (Gao et al., 2012). The significant effect of inorganic fertilizer treatments on P and K concentration in sweet potato vines and their subsequent effect on growth and yield were due to the increased availability of the nutrients in the soil solution. Root length and spatial availability are of high importance for nutrients such as P, K, and Zn (Marschner, 1995).

The attainable yield of NASPOT 11 is ~45–48 t ha^−1^ (Mwanga et al., 2011) while the highest attained yield was 34.6 t ha^−1^ from N0.25PK treatment (15 kg P ha^−1^). Therefore, application of N0.25PK can alleviate yield constraints by 74%. Although there were significant improvements by some of the treatments on growth and yield, there was still a yield gap of 11.9 t ha^−1^ for the cultivar which could be linked to a consortium of complex interacting factors. Therefore, there is a need to screen for efficient AMF strains and dosage that could improve nutrient acquisition and test their effect on sweet potato growth and yield when combined with NK and varying rates of soluble P. Abdel-Razzak et al. (2013) reported improved sweet potato growth and yield response to AMF (*G. mosseae*) combined with superphosphate fertilizer compared with single use of either input after 120 days of growth. However, the initial soil available P was high (23–25 mg kg^−1^) unlike in this study at 1.54–3.76 mg kg^−1^. Sastry et al. (2000) reported significant improvement in AMF root colonization of *Eucalyptus hybrid* and subsequent growth and P uptake when inoculum was combined with >20 mg P kg^−1^, but they all decreased when the P rate was increased to 30 mg P kg^−1^. This and other reports from Rakshit and Bhadoria (2008) and Schubert and Hayman (1986) reveal that there's no standard level of available P for realizing the greatest AMF benefits. However, available P of between >20 and 150 mg kg^−1^ promotes AMF root colonization and its subsequent effect on growth and yield. This study has demonstrated that AMF biofertilizer may not be suitable for sweet potato when applied singly especially in nutrient poor soils. Therefore, there's need of reviewing their application rates and combining them with limiting nutrients depending on soil analysis results.

The positive tuber yield response to AMF inoculation in the Ferralsol (sandy-loam) with low organic carbon (1.25%) during the short-rain season indicates the possibility that the mycorrhizal inoculation improved water use efficiency (WUE) of sweet potato. Arbuscular mycorrhizal fungi root colonization increases with water stress and this affects plant-soil water relations under drought which have an impact on physiological processes such as photosynthesis rate (Auge, 2001). Relatedly, Johnson et al. (2010) reported that shifts in environmental conditions like rainfall patterns modify mycorrhizal response. However, further investigation is required to confirm AMF beneficial effect in drought conditions in sweet potato. The uneven distribution and intensity of rainfall at the two sites during the two seasons (Figures 2A,B) contributed to lower than the potential yield by affecting tuber expansion that occurs from 10 weeks after planting (Traynor, 2005). Tuber yield levels were however larger ranging from 12.8 to 20.1 t ha^−1^ in the Rhodic Nitisol (sandy-clay) compared to 7.6 to 14.9 t ha^−1^ in the Ferralsol (sandy-loam) during the same season. This is possibly due to the positive influence of vine and leaf growth rate hence the increased supply of photosynthates resulting into sucrose synthesis and tuber development (Mengel et al., 2001).

### Soil type and seasonal variability

Arbuscular mycorrhizal fungi colonization has been previously shown to increase with water stress (Auge, 2001) which may be one of the reasons why the AMF root colonization increased during the short-rain season (Figures 2A,B). The increased colonization had a positive influence on the concentration of P and Zn in the sweet potato vines suggesting the contribution of AMF to nutrient solubilization and extended rhizosphere for nutrient acquisition (Parewa et al., 2010). Nutrient uptake increases with increased moisture content since high moisture changes the availability of nutrients (Misra and Tyler, 1999). Increased moisture content during the long-rain season significantly boosted most of the treatments in promoting the uptake of P (Figure 4A) and Zn (Figure 4D). However, P concentration was higher in the Ferralsol than in the Rhodic Nitisol which reflects the initial soil available P (3.76 and 1.54 mg kg^−1^, respectively) (Figure 4B and Table 2). The total carbon (T.C) in the soils also affected moisture availability which in turn influenced K uptake. For Rhodic Nitisol (sandy-clay), significant effects of treatments were observed in both seasons due to high T.C (3.21%) hence an improved water holding capacity of 0.31 cm^3^ water/cm^3^ soil. The Ferralsol (sandy-loam) had a lower T.C content (1.25%) hence a lower water holding capacity of 0.19 cm^3^ water/cm^3^ soil. This only promoted significant effect of the biofertilizer treatments in the short-rain season where there was increased AMF root colonization and improved moisture mobilization that positively influenced K uptake. Water deficits reduce leaf water potential and total water use, and subsequently reduce stomatal conductance, leaf area, root mass, tuber development, and total plant mass (Schneider et al., 1993). The influence of high moisture content on nutrient uptake during the long-rain season positively affected the root growth and yield leading to the seasonal differences (Figures 5B–D).

## Conclusion

The fertility of the soils used in this study ranged from low to medium which confirms the importance of biofertilizer/fertilizer use in bridging sweet potato yield gap. In these low/medium fertility soils, there was a response to applied P and K hence their improved uptake and boosting of Zn uptake. Significant effect of biofertilizer and NPK observed on yield during the short-rain season in the Ferralsol indicates the possibility that mycorrhizal inoculation improved WUE of sweet potato. It also shows the responsiveness of the Ferralsol to NPK fertilizer application. The increased AMF root colonization in the short-rain season confirms that colonization increases with water stress and that significant yield response is due to the effect of extended rhizosphere through fungal hyphae on nutrient and water acquisition. The results also highlight the importance of AMF applied singly or in combination with N and K in enhancing sweet potato growth and yield. The significance of interacting biofertilizers with N and K suggests the need for starter nutrients for AMF hyphal growth and root colonization.

There is a need to screen for efficient AMF strains that could improve nutrient acquisition and test their effect on sweet potato growth and yield when combined with NK and varying rates of soluble P. This will be addressed in our future work on AMF mechanisms of action using different strains and varying rates of P. Low sweet potato growth and yield response to both AMF and NPK indicates the need for further investigation on other limiting conditions to bridge the yield gap in sweet potato production in Uganda. Interaction of AMF inoculation with reduced rates of inorganic fertilizers tailored to results of soil analysis can increase their effect on sweet potato growth and yield. Therefore, assessing the performance of the AMF strains in the context of integrated soil fertility management program based on a comprehensive diagnosis of limiting agro-climatic conditions is a prerequisite.

## Author contributions

RM conceived and conducted the research, collected and analyzed the data, and co-wrote the manuscript; JT and PE assisted with conceiving the research and reviewed the manuscript; AA reviewed the manuscript; MT reviewed the manuscript and CM assisted with analyzing the data and reviewed the manuscript. All authors gave a final approval of the version to be published and agreed to be accountable for all aspects of the work.

### Conflict of interest statement

The authors declare that the research was conducted in the absence of any commercial or financial relationships that could be construed as a potential conflict of interest. The handling Editor declared a shared affiliation, though no other collaboration, with one of the authors AHA and states that the process nevertheless met the standards of a fair and objective review.

## References

[B1] Abdel-Razzak H. S. Moussa A. G. Abd-El-Fattah M. A. El-Morabet G. A. (2013). Response of sweet potato to integrated effect of chemical and natural phosphorus fertilizer and their levels in combination with mycorrhizal inoculation. J. Biol. Sci. 13, 112–122. 10.3923/jbs.2013.112.122

[B2] Anderson J. M. Ingram J. S. I. (1989a). TSBF: A Handbook of Methods of Analysis Wallingford, UK: CAB International 38–39.

[B2a] Anderson J. M. Ingram J. S. I. (1989a). TSBF: A Handbook of Methods of Analysis. Wallingford, UK: CAB International, 38–39.

[B3] Anderson J. M. Ingram J. S. I. (1993a). Tropical Soil Biology and Fertility: A Handbook of Methods. Wallingford, UK: CAB International, 35.

[B3a] Anderson J. M. Ingram J. S. I. (1993b). Tropical Soil Biology and Fertility: A Handbook of Methods. Wallingford, UK: CAB International, 37.

[B4] Aritua V. Gibson R. W. (2002). The perspective of sweet potato chlorotic stunt virus in Sweet potato production in Africa: a review. Afr. Crop Sci. J. 10, 281–310. 10.4314/acsj.v10i4.27531

[B5] Auge R. M. (2001). Water relations, drought and vesicular-arbuscular mycorrhizal symbiosis. Mycorrhiza 11, 3–42. 10.1007/s005720100097

[B6] Bailey J. S. Ramakrishna A. Kirchhof G. (2008). An evaluation of nutritional constraints on sweet potato (*Ipomoea batatas*) production in the central highlands of Papua New Guinea. Plant Soil 316, 97–106. 10.1007/s11104-008-9762-6

[B7] Bourke M. (2009). Sweet potato in Oceania, in Loebenstein, ed Thottappilly G. (Berlin: The Sweet Potato Springer), 489–502.

[B8] Bourke R. M. (2005). The continuing Ipomoea revolution in Papua New Guinea, in The Sweet Potato in Oceania: A Reappraisal Ethnology Monographs 19, eds Ballard C. Brown P. Bourke R. M. Harwood T. (Pittsburgh, PA; Sydney, NSW: Department of Anthropology, University of Pittsburgh; Oceania Monograph 56 The University of Sydney), 171–180.

[B9] Bouyoucos G. J. (1962). Hydrometer method improved for making particle size analyses of soils. Agron. J. 53, 464–465. 10.2134/agronj1962.00021962005400050028x

[B10] Bünemann E. Bossio D. Smithson P. Frossard E. Oberson A. (2004). Microbial community composition and substrate use in a highly weathered soil as affected by crop rotation and P fertilization. Soil Biol. Biochem. 36, 889–901. 10.1016/j.soilbio.2004.02.002

[B11] Cardoso I. M. Kuyper T. W. (2006). Mycorrhizas and tropical soil fertility. Agric. Ecosyst. Environ. 116, 72–84. 10.1016/j.agee.2006.03.011

[B12] Ceballos I. Michael R. Fernández C. Penã R. Rodríguez A. Sanders I. R. (2013). The *in-vitro* mass-produced model mycorrhizal fungus, *Rhizophagus irregularis*, significantly increases yields of the globally important food security crop cassava. PLoS ONE 8:e70633. 10.1371/journal.pone.0070633 23950975 PMC3737348

[B13] International Potato Centre. (CIP) (2006). The Use of Orange-Fleshed Sweet Potato to Combat Vitamin A Deficiency in Uganda: A Study of va. ietal Preferences, Extension Strategies and Post-Harvest Utilization. Social Sciences Working Paper No. 2006–2.

[B14] Cook A. M. (1988). Combined carbon and phosphorus or carbon and sulphur substrates, in Mixed and Multiple Feedstocks, eds Hamer G. Egli T. Snozzi M. (Konstanz: EFB), 71–83.

[B15] Estaun V. Camprubi A. Joner E. J. (2002). Selecting arbuscular mycorrhizal fungi for field application, in Mycorrhizal Technology in Agriculture, eds Gianinazzi S. Schuepp H. Barea J. M. Haselwandter K. (Berlin: Birkhauser Verlag), 249–259.

[B16] Fattah O. A. (2013). Effect of Mycorrhiza and phosphorus on micronutrients concentration by soybean plant grown in acid soil. Intern. J. Agron. Plant Prod. 4, 429–437. Available online at: http://www.ijappjournal.com/wp-content/uploads/2013/03/429-437.doc.pdf

[B17] FAO (2002). Food and Agricultural Organization. (FAO) Statistics Food and Agriculture Organization, Rome. Available online at: http://www.fao.org/statistics/en/

[B18] Gao X. Hoffland E. Stomph T. Grant C. A. Zou C. Zhang F. (2012). Improving zinc bioavailability in transition from flooded to aerobic rice. A review. Agron. Sustain. Dev. 32, 465–478. 10.1007/s13593-011-0053-x

[B19] Hajiboland R. Aliasgharzad N. Barzeghar R. (2009). Influence of arbuscular mycorrhizal fungi on uptake of Zn and P by two contrasting rice genotypes. Plant Soil Environ. 55, 93–100.

[B20] Ingleby K. (2007). Assessment of Mycorrhizal Diversity in Soils and Roots, and Nursery Inoculation to Improve the Survival and Growth of Seedlings. Mycorrhizal Training Manual.

[B21] INVAM (2017). International Culture Collection of (Vesicular) Arbuscular Mycorrhizal Fungi. West Virginia University, Morgantown, West Virginia Available online at: http://invam.wvu.edu/the-fungi/species-descriptions

[B22] Jenkins W. R. (1964). A rapid centrifugal flotation technique for separating nematodes from soil. Plant Dis. Res. 48, 692.

[B23] Johnson N. C. Wilson G. W. Bowker M. A. Wilson J. A. Miller R. M. (2010). Resource limitation is a driver of local adaptation in mycorrhizal symbioses. Proc. Natl. Acad. Sci. U.S.A. 107, 2093–2301. 10.1073/pnas.0906710107 20133855 PMC2836645

[B24] Kapinga R. Ortiz O. Ndunguru J. Omiat E. Tumwegarime S. (2007). Hand Book of Sweet Potato Integrated Crop Management, Research Outputs and Programs for Eastern Africa (1995-2006). Kampala: International Potato Centre (CIP).

[B25] Kapulnik Y. Lahkim L. T. L. Zipori I. Hazanovsky M. Wininger S. Dag A. (2010). Effect of AMF application on growth, productivity and susceptibility to *Verticillium wilt* of olives grown under desert conditions. Symbiosis 52, 103–111. 10.1007/s13199-010-0085-z

[B26] Kavoo-Mwangi A. M. Kahangi E. M. Ateka E. Onguso J. Jefwa J. M. (2014). Commercial microbiological products affect nutrient concentration of tissue cultured banana in three soil types in Kenya. Int. J. Agrisci. 4, 344–355.

[B27] Kavoo-Mwangi A. M. Kahangi E. M. Ateka E. Onguso J. Mukhongo R. W. Mwangi E. K. (2013). Growth effects of microorganisms based commercial products inoculated to tissue cultured banana cultivated in three different soils in Kenya. Appl. Soil Ecol. 64, 152–162. 10.1016/j.apsoil.2012.12.002

[B28] Kirchlof J. (2009). Soil Fertility in Sweet Potato Based Cropping Systems in the Highlands of New Papua Guinea. ACIAR Technical Report Series, No. 71, ACIAR, Canberra, 126.

[B29] Koske R. E. Gemma J. N. (1989). A modified procedure for staining roots to detect VA mycorrhizas. Mycol. Res. 92, 486–489. 10.1016/S0953-7562(89)80195-9

[B30] Lambers H. Finnegan P. M. Laliberté E. Pearse S. J. Ryan M. H. Shane M. W.. (2011). Phosphorus nutrition of *Proteaceae* in severely phosphorus-impoverished soils: are there lessons to be learned for future crops? Plant Physiol. 156, 1058–1066. 10.1104/pp.111.174318 21498583 PMC3135942

[B31] Lambers H. Raven J. A. Shaver G. R. Smith S. E. (2008). Plant nutrient-acquisition strategies change with soil age. Trends Ecol. Evol. 23, 95–103. 10.1016/j.tree.2007.10.008 18191280

[B32] Marin M. (2006). Arbuscular mycorrhizal inoculation in nursery practice, in Handbook of Microbial Biofertilizers, ed Rai M. K. (Lucknow: International Book Distributing Co.), 289–324.

[B33] MAAIF (1992). Report on Uganda National Census of Agriculture and Livestock (1990–1991), I. Methodology of the Census, II: Holding Characteristics, III: Crop Area, Yield and Production. Ministry of Agriculture Animal Industries and Fisheries. (MAAIF), Entebbe, Uganda.

[B34] Marschner H. (1995). Mineral Nutrition of Higher Plants, 2nd Edn. London: Academic Press.

[B35] McGonigle T. P. Miller M. H. Evans D. G. Fairchild G. L. Swan J. A. (1990). A new method which gives an objective measure of colonization of roots by vesicular-arbuscular mycorrhizal fungi. New Phytol. 115, 495–501. 10.1111/j.1469-8137.1990.tb00476.x 33874272

[B36] Mengel K. Kirkby E. Kosegarten H. Appel T. (2001). Principles of Plant Nutrition. 5th Edn. Dordrecht: Kluwer Academic Publishers.

[B37] Mia M. A. B. Shamsuddin Z. H. Zakaria W. Marziah M. (2002). Plant growth promoting rhizobacteria for banana production under hydroponics condition, in Sustainable Land Management, eds Shamshuddin J. Hamdan J. Samsuri A. W. (Kuala Lumpur: Malaysian Society of Soil Science), 185–190.

[B38] Misra A. Tyler G. (1999). Influence of soil moisture on soil solution chemistry and concentrations of minerals in the calcicoles *Phleum phleoides* and *Veronica spicata* grown on a limestone soil. Ann. Bot. 84, 401–410. 10.1006/anbo.1999.0941

[B39] Mukhongo R. W. Tumuhairwe J. B. Ebanyat P. Abdelgadir A. H. Thuita M. Masso C. (2016). Production and use of Arbuscular Mycorrhizal Fungi Inoculum in Sub-Saharan Africa: challenges and ways of improving. Int. J. Soil Sci. 11, 108–122.

[B40] Mwanga R. O. M. Niringiye C. Alajo A. Kigozi B. Namukula J. Mpembe I. (2011). “NASPOT 11,” a sweet potato bred by a participatory plant-breeding approach in Uganda. HortScience 46, 317–321.

[B41] Mwanga R. O. M. Odongo B. Ocitti p'Obwoya C. Turyamureba G. M. (2001). Sweet potato (*Ipomoea batatas* (L.) Lam.), in Agriculture in Uganda, Vol. II, Crops, ed Mukiibi J. K. (Kampala: National Agricultural Research Organisation (NARO)-CTA. Fountain Pub), 233–251.

[B42] O'Sullivan J. N. Asher C. J. Blamey F. P. C. (1997). Nutrient Disorders of Sweet Potato. Canberra, CT: ACIAR Monograph Series.

[B43] Okalebo J. R. Gathua K. W. Woomer P. L. (2002). Laboratory Methods for Soil and Plant Analysis. A Working Manual. 2nd Edn. Tropical soil fertility and Biology Program, Nairobi Kenya TSBF-CIAT and SACRED Africa, Nairobi, Kenya.

[B44] Ortas I. (2010). Effect of mycorrhiza application on plant growth and nutrient concentration in cucumber production under field conditions. Span. J. Agric. Res. 8, S116–S122. 10.5424/sjar/201008S1-1230

[B45] Osiru M. O. Olanya O. M. Adipala E. Lemaga B. Kapinga R. (2009). Stability of Sweet potato cultivars to *Alternaria* leaf petiole and stem blight disease in diverse environments. J. Phytopath. 157, 172–180. 10.1111/j.1439-0434.2008.01457.x

[B46] Parewa H. P. Rakshit A. Rao A. M. Sarkar N. C. Raha P. (2010). Evaluation of maize cultivars for phosphorus use efficiency in an Inceptisol. I. J. Agric. Environ. Biotech. 3, 195–198.

[B47] Pender J. Jagger P. Nkonya E. (2004). Development pathways and land management in Uganda. World Dev. 32, 767–792. 10.1016/j.worlddev.2003.11.003

[B48] Porras-Soriano A. Meddad-Hamzal A. Beddiar A. Gollotte A. Lemoine M. C. Kuszala C. (2010). Arbuscular mycorrhizal fungi improve the growth of olive trees and their resistance to transplantation stress. Afr. J. Biotechnol. 9, 1159–1167. 10.5897/AJB09.1282

[B49] PRAPACE (2003). Regional Potato and Sweet Potato Improvement Programme in East and Central Africa, Five-Year Priority Setting. PRAPACE, 2003–2008. PRAPACE, Kampala, Uganda.

[B50] Radhika K. P. Rodrigues B. F. (2010). Arbuscular mycorrhizal fungal diversity in some commonly occurring medicinal plants of western Ghats, goa region. J. Forestry Res. 21, 45–52. 10.1007/s11676-010-0007-1

[B51] Rakshit A. Bhadoria P. B. S. (2008). Indigenous arbuscular mycorrhiza is more important for early growth period of groundnut (*Arachis hypogea* L.) for P influx in an Oxisol. Acta Agric. 91, 397–406. 10.2478/v10014-008-0020-7

[B52] Rhoades J. D. (1982). Cation exchange capacity, in Methods of Soil Analysis, Part 2. Chemical and Microbiological Properties, eds Page A. L. Miller R. H. Keeney D. R. (Madison, WI: American Society of Agronomy, Inc.; Soil Science Society of America. Inc.), 149–157.

[B53] Rosendahl S. (2008). Communities, populations and individuals of arbuscular mycorrhizal fungi. New Phytol. 178, 253–266. 10.1111/j.1469-8137.2008.02378.x 18248587

[B54] Sastry M. S. R. Sharma A. K. John B. N. (2000). Effect of an AM fungal consortium and Pseudomonas on the growth and nutrient concentration of Eucalyptus hybrid. Mycorrhiza 10, 55–61. 10.1007/s005720000057

[B55] Schenck N. C. Perez Y. (1990). Manual for Identification of VA Mycorrhizal Fungi. Gainsville, FL: Synergistic Publications.

[B56] Schneider J. Widyastuti C. A. Djazuli M. (1993). Sweet Potato in the Baliem Valley Area, Irian Jaya. In a report on collection and study of sweet potato germplasm, April-May 1993. International Potato Center (CIP), ESEAP-Region; Bogor, Indonesia.

[B57] Schubert A. Hayman D. S. (1986). Plant growth responses to vesicular–arbuscular mycorrhiza XVI. Effectiveness of different endophytes at different levels of soil phosphate. New Physiol. 103, 79–90. 10.1111/j.1469-8137.1986.tb00598.x

[B58] Sharma S. B. Sayyed R. Z. Trivedi M. H. Gobi T. A. (2013). Phosphate solubilizing microbes: sustainable approach for managing phosphorus deficiency in agricultural soils. SpringerPlus 2:587. 10.1186/2193-1801-2-587 25674415 PMC4320215

[B59] Smith F. W. Mudge S. R. Rae A. L. Glassop D. (2003). Phosphate transport in plants. Plant Soil 248, 71–83. 10.1023/A:1022376332180

[B60] Taraken I. T. Kapal D. Sirabis W. Bailey J. (2010). Nutrient deficiencies limiting the growth of sweet potato vines on important soil types in the Highlands of New Papua Guinea, in 19^th^ World Congress of Soil Science, Soil Solutions for a Changing World (Bribane).

[B61] Traynor M. (2005). Sweet Potato Production Guide for the Top End. Information Booklet 1. Department of Primary Industry, Fisheries and Mines Crops, Forestry and Horticulture Division Northern Territory Government. Available online at: www.horticulture.nt.gov.av

[B62] Uwah D. F. Undie U. L. John N. M. Ukoha G. O. (2013). Growth and yield response of improved sweet potato (*Ipomoea batatas* (L.) *Lam*) varieties to different rates of potassium fertilizer in Calabar, Nigeria. J. Agric. Sci. 5, 61–69. 10.5539/jas.v5n7p61

[B63] Vanlauwe B. Bationo A. Chianu J. Giller K. E. Mercks R. Mokwunye U. (2010). Integrated soil fertility management: operational definition and consequences for implementation and dissemination. Outlook Agric. 39, 17–24. 10.5367/000000010791169998

[B64] Verbruggen E. van der Heijden M. G. Rillig M. C. Kiers E. T. (2012). Mycorrhizal fungal establishment in agricultural soils: factors determining inoculation success. New Phytol. 197, 1104–1109. 10.1111/j.1469-8137.2012.04348.x 23495389

[B65] Wang C. Li X. Zhou J. Wang G. Dong Y. (2008). Effects of arbuscular mycorrhizal fungi on growth and yield of cucumber plants. Comm. Soil Sci. Plant Anal. 39, 499–509. 10.1080/00103620701826738

[B66] Yost D. Eswaran H. (1990). Major Land Resource Areas of Uganda. Washington, DC: Soil Management Servives, USAID, 218.

